# Effect of promoter type and synthesis method on catalytic performance of Fe-Mn based FT-olefin catalysts

**DOI:** 10.55730/1300-0527.3406

**Published:** 2022-04-28

**Authors:** Gamze GÜMÜŞLÜ GÜR, Özge ATİK

**Affiliations:** 1 Department of Chemical Engineering, Faculty of Chemical and Metallurgical Engineering, İstanbul Technical University, İstanbul, Turkey; 2 İTU Synthetic Fuels and Chemicals Technology Center, İstanbul Technical University, İstanbul, Turkey

**Keywords:** Fischer-Tropsch synthesis, light olefins, Fe-Mn-Cu-K, n-pentane, solvent, high-throughput

## Abstract

Direct production of light olefins, building blocks of chemical industry, can be attained by developing efficient catalysts for Fischer–Tropsch synthesis (FTS). The nature of FTS complicates the catalyst development process as the product distribution is affected by the components and the preparation methods of the catalyst. In this work, high-throughput (HT) methodology is employed to overcome this problem by testing many different catalyst formulations. Fast performance screening of 40 different α-Al_2_O_3_ supported Fe-Mn based catalysts promoted with Cu, K and Ni, using different impregnation agents, was performed in a HT test system at atmospheric pressure. Promising catalyst candidates identified by HT analysis were further subjected to high pressure FTS in a conventional system. Results indicate that coupled with Mn, Ni promoted CH_4_ production, Cu increased CO conversion, K enhanced olefin selectivity and olefin-to-paraffin ratio. In double promotion of Cu and K, Cu balanced the activity and stability loss due to K, while K enhanced olefin selectivity. *n*-pentane aided impregnation slightly enhanced catalytic performance. Differences observed in catalytic performance were regarded as related to the structural changes caused by promoter and impregnation type based on characterization data obtained by H_2_-TPR, XRD, SEM, EDS mapping and N_2_ adsorption.

## 1. Introduction

Production of most of the chemical commodities used in industry or daily life depends on petroleum derived raw materials [1]. As the availability of petroleum and the carbon footprint of petroleum based processes are debatable, alternative routes for production of chemical commodities have been researched. Among those, light olefins (LOs) production stands out as a good example. LOs are raw materials for various critical chemical compounds such as PET, ethanol, acrylonitrile, adhesives [2]. However, the commercial production method of LOs relies on naphtha which is derived from petroleum [3]. Methods of direct production from syngas (CO+H_2_) have been at the forefront of research on developing a more environmental-friendly LOs production process [4,5]. Syngas can be obtained from biomass, waste or coal paving the way for decreased carbon footprint of the process.

Fischer–Tropsch synthesis (FTS) offers a solution to the problem of finding a direct synthesis method of LOs from syngas [1,6]. FTS has a polymerization-like mechanism in which CH_x_ radicals come together to form short/long chain hydrocarbons (HC) [7]. Therefore, FTS leads to a wide range of products [8]. To restrain the FTS products in LOs range, it is required to design an efficient and selective catalyst and, as such, achieve high CO conversion and LOs selectivity [9,10].

Typical active metals used in FTS, namely Fe, Co, Ni and Ru, lead to different product selectivities [10]. For FT-olefin synthesis, Fe is generally the choice of active metal due to its lower cost and higher capability of suppressing methane and heavy HCs [1,11]. Unfortunately, Fe alone is not enough to achieve high LOs selectivity. Use of promoters enhance the performance of Fe catalysts by altering the reducibility, dispersion or interaction of active sites [12]. Mn, as a structural promoter, is known to increase active site dispersion and decrease secondary hydrogenation of alkenes [11,13]. However, higher concentrations of Mn decreases the reducibility and carburization of Fe due to the strong interaction of Mn with Fe [11,14]. Therefore, it is crucial to precisely specify the correct amount of Mn. Cu, as a transition metal, is used to promote catalyst activity and stability. The increase in activity is related to the fact that Cu enhances iron carbide formation and reducibility of Fe oxides [15–17]. Similarly, Ni promoter is observed to increase CO conversion by enhancing Fe oxide dispersion [17,18]. Although, Ni is generally preferred as active metal in FTS for production of CH_4_, as a promoter in Fe catalyst, it yields different product distributions, and even an increase in light olefins production [19–21]. K, an alkali promoter, suppresses the production of paraffins and methane, and increases olefin/paraffin ratio [22–24]. However, high amounts of K inhibit the reduction of iron oxides and decrease the activity covering the catalyst active sites [25, 26]. Therefore, using Mn, Cu, K and Ni promoters with the right amount might increase the activity and selectivity of Fe catalyst in FT-olefin synthesis.

Another parameter affecting the FT catalyst performance is the synthesis method [16]. Effects of parameters such as calcination temperature, impregnation type (co- or sequential), precursor type on FTS activity and product distribution have been widely investigated [27–29]. However, the literature on investigating the effect of solvent in impregnation for FTS catalyst synthesis is scarce [30]. In a study by Zhang et al. [31], higher activity, stability and active metal dispersion was achieved on the Co catalysts prepared by ethanol aided impregnation. Using ethanol as impregnation agent is also reported to reduce Co oxide crystallite size [32]. Thus, it might be possible to tune the activity and selectivity of FTS catalysts through solvent-aided impregnation.

In this study, we prepared 40 Fe-based catalysts that were α-Al_2_O_3_ supported and Mn, Cu, K, Ni promoted using pentane aided and aqueous impregnation. FT-olefin performance of catalysts was first tested in our high-throughput catalyst performance analysis test system (HT-CPA) under atmospheric pressure. HT-CPA analysis results were used to identify the promising promoters and their concentrations. Subsequently, promising catalyst were tested in our pressurized test system (P-CPA) to observe their performance under realistic FT conditions. To understand catalytic behavior, catalysts were characterized by X-ray diffraction (XRD), H_2_-temperature programmed reduction (H_2_-TPR), N_2_ adsorption and energy-dispersive X-ray spectroscopy (EDS) mapping analyses.

## 2. Materials and methods

### 2.1. Catalyst preparation

All catalysts comprising Fe as active metal, Mn as first promoter and either of Cu, Ni or K as second/third promoter were synthesized using coimpregnation method. Active metal and promoters were coimpregnated on α-Al_2_O_3_ (Alfa Aesar, 99.9%) support. Support, Fe(NO_3_)_3_.9H_2_O (Merck, ACS), Mn(NO_3_)_2_.4H_2_O (Merck, >98.5%) and either of Cu(NO_3_)_2_.3H_2_O (Merck, >99.5%), Ni(NO_3_)_2_.6H_2_O (Merck, ACS) or KNO_3_ (Merck, ISO) were first taken into an Erlenmeyer flask. Then, this powder mixture was slurried either in deionized water or n-pentane (Sigma-Aldrich, reagent grade, 98%). Aqueous mixture was mixed in an ultrasonic bath, whereas n-pentane comprising solution was stirred overnight. Prepared catalysts were dried at 110 °C and calcined at 430 °C under dry air atmosphere for 3 h. Catalyst were named according to the type and amount of metal used, and the type of solvent as follows: *x***Fe***y***Mn***z****A****k****B***-*s* where *x, y, z* and *k* are the weight percentage of the metal used, *A* and *B* represent the second and third promoter (Cu, Ni or K), *s* indicates the type of solvent used (w-water, p-pentane). In this study, the weight percentage of Fe (*x*) and Mn (*y*) in the catalysts were determined to be 15 and 0.3, respectively; *z* and *k* were used as 0.5, 1, 1.5, 2, and 2.5. A representative notation for a catalyst comprising 0.3 wt.% Mn, 0.5 wt%Cu prepared by n-pentane is: *15Fe0.3Mn0.5Cu-p*.

### 2.2. Catalyst characterization

Surface area, average pore size and pore volume of the fresh (calcined) catalysts were determined by BET analysis in a Micromeritics 3 Flex Surface Characterization Analyzer. For analysis, samples were outgassed at 150 °C for 24 h under vacuum condition, and then they were subjected to N_2_-adsorption at 77 K. Five adsorption points in the relative pressure range of 0.05 to 0.30 were used in determining BET surface areas.

Morphology of the fresh (calcined) and the spent catalysts were observed through SEM (JEOL JSM-6390LV) analysis. Crystalline phases of the catalyst in fresh (calcined) and spent form were analyzed by XRD (SHIMADZU XRD-600). XRD characterization of the samples were performed in the 2θ range of 2–70° using Cu Kɑ radiation (λ = 0.154 nm). Carbon mapping of the spent catalysts was performed by EDS analysis (Zeiss EVO LS 10).

H_2_-temperature programmed reduction (H_2_-TPR) profiles of catalysts were obtained in pressurized catalyst performance analyzer (P-CPA) system operating at atmospheric pressure. For H_2_-TPR experiments, 1 g catalyst was placed into the fixed bed reactor and then heated to 350 °C under N_2_ atmosphere to purge the system. After 30 min at 350 °C, the system was cooled down to 50 °C and 10% H_2_/90% N_2_ (v/v) mixture was fed to the reactor. The sample was then heated to 800 °C at a rate of 10 °C/min. Amount of H_2_ adsorbed was measured by a micro GC with a thermal conductivity detector (TCD) (micro-GC Fusion, Inficon) and a mass spectrometer (Stanford Research Systems, RGA 200), simultaneously.

### 2.3. High-throughput catalyst performance analysis

**Figure 1:**
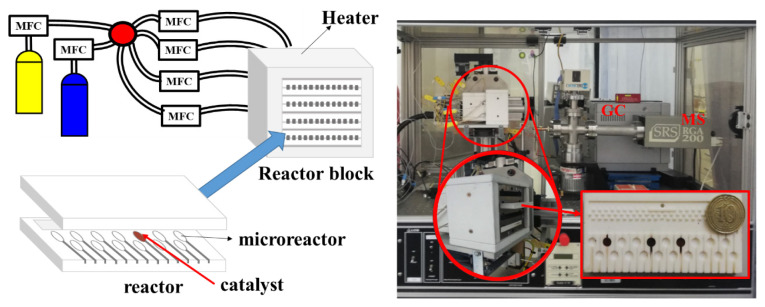
The schematic description (on the left) and the picture of HT-CPA

The prepared catalysts were initially scanned for their FT-olefin performance at atmospheric pressure in a high-throughput catalyst performance analyzer (HT-CPA), shown in Figure 1. HT-CPA, described in detail elsewhere [33], is a computer integrated high-throughput catalyst screening system that enables fast screening of multiple (max. 80) catalysts simultaneously. Reactant gases are fed to the system through two sets of calibrated mass flow controllers. First set is for mixing the gases, whereas the second set is required for separating the flow to be fed into 4 reactors in the reactor block. Each reactor is comprised of 20 microreactors, each of which receive equally distributed gas flow and require c.a. 20 mg catalyst. Outlet gases from the microreactors are sampled using a capillary sampling probe and sent directly to an on-line micro-Gas Chromatograph (micro-GC Fusion, Inficon), comprising RT-Q Bond and RT-Molecular Sieve 5A models equipped with thermal conductivity detectors (TCD). Temperature, inlet flow rates, gas sampling and movement of the reactor in HT-CPA system are controlled via an automated software.

Reduction of the catalysts was performed in HT-CPA system, prior to reaction. Catalysts were subjected to a flow of 50% H_2_ (Linde, 99.999%)/50% N_2_ (Linde, 99.999%) (v/v) mixture at 350 °C for 4 h. Reactant gas mixture composed of 60% H_2/_30% CO (Linde, 95% CO – 5% N_2_ (v/v))/10% N_2_ (v/v/v) was fed to the system after reactor was cooled to the reaction temperature of 310 °C under inert N_2_ flow.

### 2.4. Pressurized catalytic performance analysis

Analysis in HT-CPA system allowed for fast screening to identify the catalyst which can display high performance under pressurized reaction conditions. To test activity and selectivity of catalysts at realistic FT-olefin conditions in pressurized catalytic performance analysis (P-CPA) system, ca. 1 g of each catalyst was prepared and mixed in a 1:1 (v/v) ratio with Quartz (Sigma-Aldrich, purum p.a.). P-CPA, which is used for the pressurized FT-olefin reaction experiments, is a conventional fixed-bed reaction system, equipped with an on-line Gas Chromatograph GC (Agilent 7280) with thermal conductivity detector (TCD) and flame ionization detector (FID). Data from GC was used to calculate the FT-olefin performance parameters of the catalysts given in the equations:


(1) $$X_{CO}=\left(\frac{F_{CO,in}-F_{CO,out}}{F_{Co,in}}\right)\times 100,$$

where *X_CO_* is the CO conversion, *F_CO,in_* and *F_CO,out_* are inlet and outlet CO molar flows.


(2) $$F_{HC\;(Cn)}=F_{total,out}\times x_{HC\;(Cn)},$$

where *F_HC (Cn)_* is the molar flow of the produced hydrocarbon with the specific carbon number, *F_total,out_* is the total molar flow at the outlet, *x_HC (Cn)_* is the mole fraction of that specific hydrocarbon.


(3) $$S_{HC(C\;basis)}=\left(\frac{n_{C}\times F_{HC\;(Cn)}}{(F_{CO,in}-F_{CO,out})-F_{CO_{2},out}}\right)\times 100,$$

where *S_HC(C basis)_* is the hydrocarbon selectivity calculated on carbon basis, *n_(C)_* is the number of carbons in the hydrocarbon, *F_H_*_*_2_*_*_,out_* is the molar flowrate of CO_2_ in the outlet.


(4) $$S_{total\;coke/HC\;C6+(C\;basis)}=100-\Sigma_{i=1}^{5}S_{HC,i},$$

where *S_total coke/HC C6+(C basis)_* represents the selectivity to coke and/or heavy hydrocarbons.


(5) $$C_{n}\%=\left(\frac{n_{C.}F\;HC\;(Cn)}{\Sigma_{i=1}^{5}n_{C}.F_{HC\;(Cn),i}}\right)\times 100,$$

where C_n_% is the hydrocarbon distribution on carbon basis.


(6) $$Olefin\;Yield=\frac{\Sigma_{i=2}^{4}n_{C}.F_{C_{2}^{=}-C_{4}^{=}}\times MW_{C}}{gFe},$$

where $F_{C_{2}^{=}-C_{4}^{=}}$ is the molar flow rate of produced $C_{2}^{=}-C_{4}^{=}$ olefins, *MW_C_* is the molecular weight of C and *gFe* is the gram Fe used in catalyst.


(7) $$FT\;Yield=\frac{\Sigma_{i=1}^{5}n_{C}.F_{HC\;(Cn)}}{gFe},$$

where FT yield is the Fischer–Tropsch yield as a measure of CO conversion to hydrocarbons.

In addition to these FT-olefin performance parameters, H_2_ to CO usage ratio was calculated for detailed comparison of catalysts:


(8) $$H_{2}/CO=\frac{F_{H_{2}.in}-F_{H_{2}.out}}{F_{CO,in}-F_{CO,out}},$$

where *F_H_*_*_2_*_*_,in_* and *F_H_*_*_2_*_*_,out_* represent the molar inlet and outlet flowrates of H_2_.

## 3. Results and discussion

### 3.1. Initial performance screening of catalysts in HT-CPA

HT-CPA system was used for fast initial screening of catalytic performances of the 40 different Fe-based catalysts using the methods described above. The analysis with HT-CPA results in a basic comparative data set that can be used to determine the promising catalysts in a much shorter time in comparison to analysis with conventional systems. Prepared catalysts and the HT-CPA analysis results are given in Table 1.

In Table 1, effect of promoter type and concentration on FT-olefin performance can be observed at atmospheric pressure. By varying the catalyst properties, the aim is to increase CO conversion, $C_{2}^{=}-C_{4}^{=}$ olefins production and selectivity; and lower CO_2_ and CH_4_ production and the selectivity to CH_4_. In FT-olefin synthesis, distribution/selectivities of hydrocarbons are of as much importance as the magnitudes of their production to understand the behavior of the catalysts in shifting product distribution towards light olefins. Therefore, in HT-CPA data analysis relative amounts of $C_{2}^{=}-C_{4}^{=}$ and CH_4_ with respect to other hydrocarbons along with their production were considered. The data set for each performance indicator, as given in Table 1, was normalized with respect to the highest and lowest values obtained in the set.

HT-CPA data (Table 1) shows that regardless of the metal, increasing the promoter concentration above 0.5 wt.% does not enhance CO conversion. Further analysis shows that use of n-pentane improved the conversion obtained on Mn-K promoted catalysts. It was also seen that Mn-K combination had a positive effect on catalyst performance in suppressing CO_2_ and CH_4_ production, and improving $C_{2}^{=}-C_{4}^{=}$ selectivity, which was more pronounced when impregnated by n-pentane. Although the production of $C_{2}^{=}-C_{4}^{=}$ was low, this was compensated by the ability of K in lowering the production of CH_4_ and paraffins [23,34]. Another observation is the negative effect of Ni on catalyst performance in increasing both the production of and the selectivity to CH_4_. This is an expected result as Ni is generally used for CH_4_ production purposes [35].

The data in Table 1 is invaluable as it displays the overview of the catalytic performances of 40 different catalysts while presenting the results in a comparative way. Nevertheless, determining the promising catalyst candidates from such data set by pure observation is challenging. Therefore, for each performance indicator data points were graded from 0 to 100 from undesired to the most desired data value. Then, the grades of each catalyst for each performance indicator were summed up to reach a final performance grade. These final grades are represented in Table 2.

According to Table 2, the top three FT-olefin performances were observed on 15Fe0.3Mn0.5Cu-p, 15Fe0.3Mn0.5Cu-w and 15Fe0.3Mn0.5K-p catalysts. These catalysts were regarded as promising catalysts candidates, and along with 15Fe0.3Mn0.5K-w were further subjected to P-CPA tests and characterization analyses to understand the effect of promoter and impregnation agent on catalyst performance. In addition to these catalysts, 15Fe0.3Mn0.5Cu0.5Ni-w, and 15Fe0.3Mn0.5Cu0.5K-w catalysts from the first raw of Table 2 were also tested in P-CPA to obtain a comprehensive data set to compare HT-CPA predictions with P-CPA results. As HT-CPA analysis only points out the candidate catalysts, the actual performance of the catalysts can be elucidated under high pressure-high temperature realistic FT conditions.

### 3.2. Pore structures and surface areas

The HT-CPA analysis results referred 15Fe0.3Mn0.5Cu-p, 15Fe0.3Mn0.5Cu-w and 15Fe0.3Mn0.5K-p catalysts as the promising candidates. Therefore, to understand the effect of Cu and K promoters and the impregnation agent on surface textures of the catalysts, these catalysts along with 15Fe0.3Mn0.5K-w were analyzed by N_2_-adsorption experiments as described earlier. Related results for each catalyst are given in Table 3.

For catalysts prepared using water, addition of Cu led to a slight increase in the BET surface area and a decrease in average pore diameter as seen in Table 3. Similar effects of Cu have also been reported in the literature [36, 37]. This behavior has been explained by Cu promotion leading to smaller crystallite formation that are well dispersed across catalyst surface [37]. The decrease in pore volume and the increase in the micropore volume also suggests that Cu might be filling mesopores such that the reduction in pore dimensions due to presence of Cu leads to transformation of mesopores into micropores. Using K promoter instead of Cu on 15Fe0.3Mn catalyst caused a significant decrease in the surface area by almost half. Although the pore volume obtained on K promoted catalyst was similar to that of on Cu promoted one, the average pore diameter was almost doubled. Similar effects of K promoter on precipitated iron catalysts were previously reported in literature and explained by the growth in crystal size due to addition of K to the catalyst [36, 37]. The drastic change in both average pore diameter and external surface area also suggests that K might be placed into the macropores on the surface creating mesopores. However, to understand the effect of K in detail, a thorough surface analysis is required.

Switching impregnation agent from water to pentane led to a decrease in the surface area of the prepared catalysts. The decrease in surface area was accompanied by a reduction in pore volume for 15Fe0.3Mn and 15Fe0.3Mn0.5Cu catalysts. Therefore, the effect of using pentane as impregnation agent can be interpreted as an enhanced diffusion of metals into the pores of the support, or as an increase in crystallite size. On the other hand, besides pore volume, no significant change was observed on the textural properties of K promoted catalyst. As described above, this could be due to the enhancing effect of K on crystal growth.

### 3.3. Surface morphology

**Figure 2:**
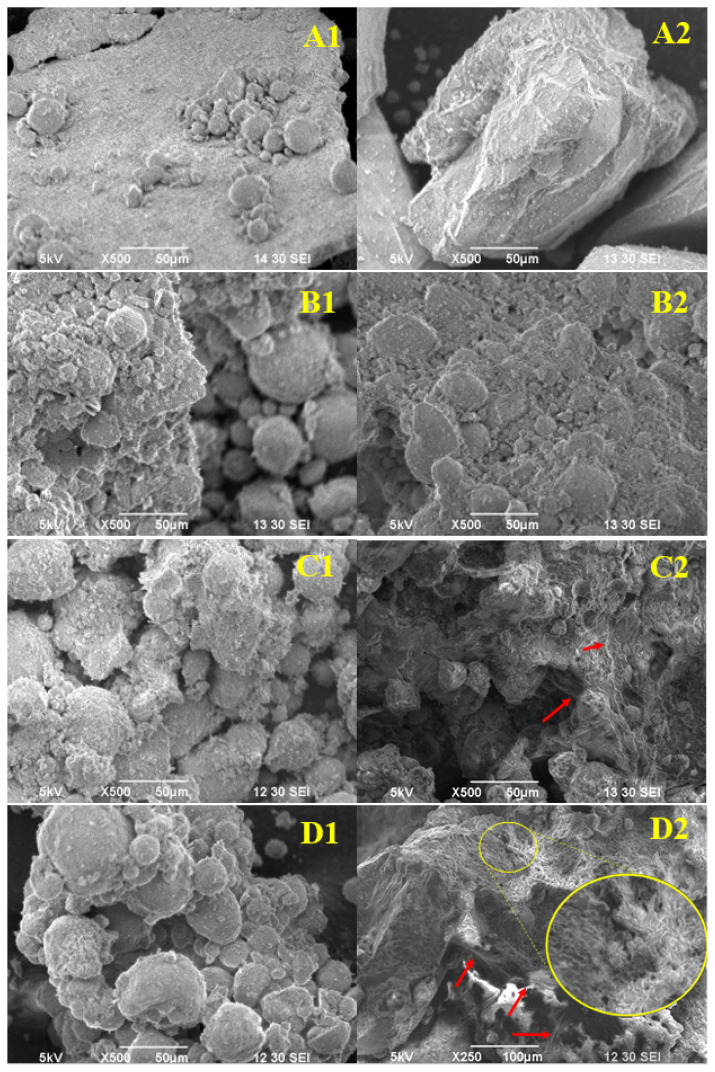
SEM images of the fresh (calcined) (1) and spent (2) 15Fe0.3Mn0.5Cu-w (A), 15Fe0.3Mn0.5Cu-p (B), 15Fe0.3Mn0.5K-w (C), and 15Fe0.3Mn0.5K-p (D) catalysts.

The effects of promoters and impregnation agents on surface morphology of catalysts were investigated via SEM images provided in Figure 2.

As seen from Figure 2, surface morphologies of fresh (calcined) catalysts were similar in appearance. However, a clear distinction existed between the surface features of the Cu and K promoted spent catalysts. On Cu promoted catalysts, regardless of the impregnation agent, no significant changes were observed between the fresh and spent catalysts, as can be inferred comparing the inset figures A1 vs. A2, B1 vs. B2 and A2 vs. B2 of Figure 2. On the other hand, fibril-like structure formation was observed on spent catalysts comprising K promoter, as shown with red arrows in Figures 2C2 and 2D2. It was also observed that this structure was more distinct in catalyst prepared by pentane aided impregnation, as identified by the zoomed in area in Figure 2D2. This fibril-like structure resembles the reported SEM images of manganese oxide (Mn_3_O_4_) and mixed Fe-Mn oxide (FeMnO_3_) crystallites [38,39] as well as carbon fibrils [40]. For identification of these structures, EDS analysis data along with SEM images were evaluated.

**Figure 3:**
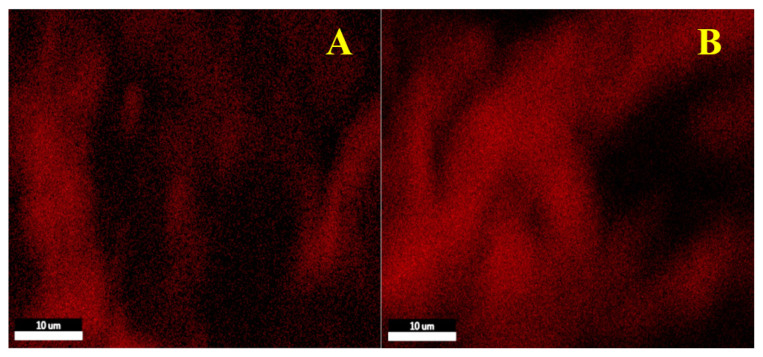
EDS C mapping of spent (A) 15Fe0.3Mn0.5K-w and (B) 15Fe0.3Mn0.5K-p catalysts.

EDS analysis of the spent 15Fe0.3Mn0.5K catalysts was performed to map C across the surface. C maps of the catalysts are given in Figures 3A and 3B. As seen from the analysis results, surfaces of both catalysts were covered with C after the reaction. Comparison of the EDS results of two catalysts reveals that presence of C is more prominent on the catalyst prepared by pentane. In addition, the C content was higher than that of Fe on the surface. Therefore, it was concluded that these fibril-like structures are due to increased carbon deposition on the surface.

### 3.4. Surface phase and crystallinity

**Figure 4:**
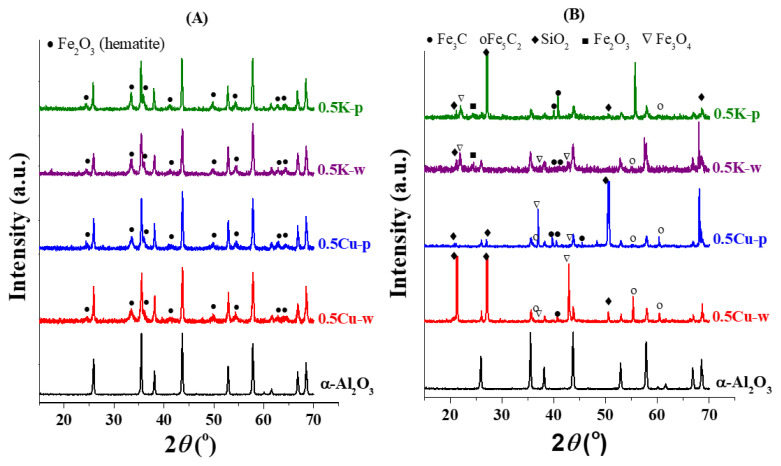
XRD profiles of the α-Al_2_O_3_ support, fresh (calcined) (A) and spent (B) catalysts. 0.5K-p, 0.5K-w, 0.5Cu-w, and 0.5Cu-p represent 15Fe0.3Mn0.5K-p. 15Fe0.3Mn0.5K-w, 15Fe0.3Mn0.5Cu-p, and 15Fe0.3Mn0.5Cu-w catalysts, respectively.

XRD profiles of fresh (calcined) and spent catalysts were evaluated to elucidate the effects of promoters and impregnation agents on crystalline phases on the catalyst surfaces. Related XRD patterns are given in Figure 4A–B.

The peaks located at 2*θ* values of 24.41°, 33.49°, 35.90°, 41.18°, 54.51°, 63.03°, and 64.52° in XRD patterns of fresh catalysts, as shown in Figure 4A, indicated formation of Fe_2_O_3_ phase upon calcination. The crystallite sizes of the Fe_2_O_3_ phase calculated based on the most intense peak at 2*θ*= 33.49° using Scherrer equation are listed in Table 4. Results in Table 4 support the conclusions inferred from N_2_ adsorption experiments in that using pentane as impregnation agent led to larger crystallites and that K promoter enhanced crystal growth.

As seen in Figure 4B, surface crystallinity and phases of catalysts changed during the course of the reaction. The iron carbide phases, which are the active phases for FT synthesis [34, 41] were detected at 2*θ* values of 39.97°, 40.81°, and 45.34° for Fe_3_C and 55.25° and 60.63° for Fe_5_C_2_, on all catalysts. While the XRD profiles of catalysts prepared with pentane displayed distinct features of Fe_3_C phase, Fe_5_C_2_ phase was more prominent on Cu promoted catalyst prepared with water. Comparing the iron carbide peak intensities, it can be concluded that use of pentane in catalyst synthesis enhanced carburization of Fe during the reaction. In addition to carbide phases, Fe_3_O_4_ phase (2*θ*= 37.03°, 43.02°) was present on all catalysts. Presence of iron oxide phase could be an indication of both the reduction of iron (from Fe_2_O_3_ to Fe_3_O_4_ phase) and the reoxidation of the carburized Fe during the reaction [42,43]. On K comprising catalysts, in addition to Fe_3_O_4_, Fe_2_O_3_ peaks were also observed. Unlike other catalysts, Fe_2_O_3_ and Fe_3_O_4_ peaks occurred at 2*θ*= 21.91° and 24.41°, respectively. These angles are also listed for Mn_3_O_4_ phase; however, the concentration of Mn is too low to be detected by XRD analysis. Nevertheless, the position of the iron oxide peaks on K comprising catalysts suggests that these oxide phases may also include contributions from dilute MnO or even Fe-Mn mixed oxide phases, because K promotor is known to enhance Fe-Mn interactions [44,45]. Formation of mixed Fe and Mn oxide phases are regarded as the enhanced selectivity to olefins in literature [13]. Furthermore, the strong interaction of Mn with Fe in presence of K promoter stabilizes metal oxide phases, as seen by XRD analysis, hindering carburization and further reduction of Fe [46]. This explains the enhanced light olefins selectivity of K promoted catalysts.

### 3.5. Reducibility of the catalysts

**Figure 5:**
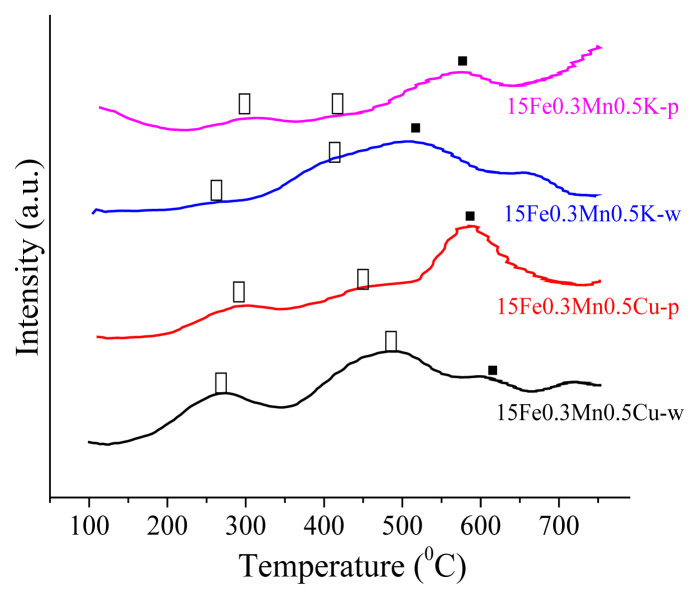
H_2_-TPR profiles of catalysts displaying reduction of (♦) (Fe, Mn)_2_O_3_ to (Fe, Mn)_3_O_4_, (●) (Fe, Mn)_3_O_4_ to (Fe, Mn)O, and (■) (Fe, Mn)O to α-Fe and MnO.

H_2_-TPR profiles, given in Figure 5, were used to investigate the reduction behavior of the catalysts. The H_2_-TPR profiles of all catalysts represent three peaks in the low, intermediate and high temperature ranges with slight changes in peak temperatures. These three peaks are assigned to the reduction of (Fe, Mn)_2_O_3_ to (Fe, Mn)_3_O_4_ (200–350 °C range), (Fe, Mn)_3_O_4_ to (Fe, Mn)O (350–550 °C range), and (Fe, Mn)O to α-Fe and MnO (550–700 °C range) [47,48]. As reported in the literature, further reduction of MnO into Mn was not observed due to thermodynamic restrictions [45].

Comparison of the first peak temperatures in Figure 5 reveals that the promoter type did not have as much influence on the reducibility of the catalyst as the impregnation agent type. It can be observed that the use of pentane slightly shifted the peak temperature of (Fe, Mn)_2_O_3_ to (Fe, Mn)_3_O_4_ reduction to a higher value for both Cu and K promoted catalysts. This could be attributed to larger Fe_2_O_3_ crystallites in catalysts prepared with pentane, because ease of reducibility decreases with the increase in crystallite size [30]. In addition, on the catalyst prepared by pentane, structures of the second and third peaks became more distinct with an increase in the tail following third peak. This feature, which was more emphasized on K promoted catalyst, indicates an enhanced active metal-support interaction with the use of n-pentane [30,49]. This difference in the degree of metal-support interaction regarding the use of water or pentane is due to the polarity difference between water (polarity index: 10.2) and pentane (polarity index: 0). The decreased polarity leads to stronger metal-support interaction [31]. Promoter type had an effect on the reduction behavior at high temperature regions. As seen in Figure 5, K promoter decreased the peak temperature of these reduction processes. This is well aligned with reported reduction behaviors of SiO_2_ supported Fe-Mn-Cu and Fe-Mn-K catalysts in the literature. Comparing the low and high temperature region peaks of Cu and K promoted catalysts from the studies by Gong, et al. [50] and Zhang et al. [51], it can be seen that although Cu promoter decreases the peak temperature related to the first reduction process, the synergistic effect of K with Mn enhances the reduction of (Fe, Mn)_3_O_4_ to (Fe, Mn)O and (Fe, Mn)O to α-Fe and MnO better than that of Cu. In addition, the decrease in the peak intensities switching from Cu to K promoter indicates the decrease in H_2_ adsorption on the surface. This is also due to the effect of K promoter suppressing H_2_ dissociation on the surface which prevents secondary hydrogenation of olefins during FT synthesis [22, 52].

### 3.6. FT-olefin performances of the catalysts

**Figure 6:**
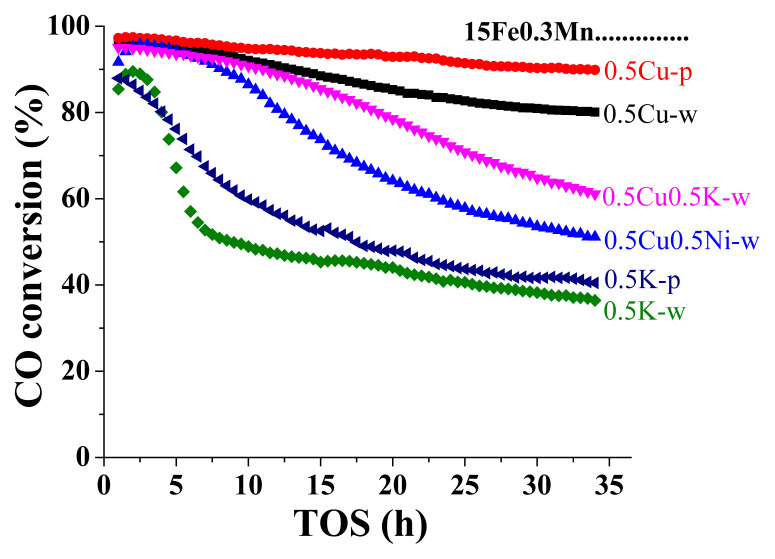
CO conversion of the 15Fe0.3Mn based catalysts versus on time on stream (TOS). Name of the catalysts prepared with different promoters and impregnation agents are provided on the figure next to each line

FT-olefin performances of the catalysts that were selected based on P-CPA results were observed at 310 °C and 10 bar under continuous supply of H_2_:CO = 2:1 gas mixture. CO conversions of catalysts, indicating their activity in FT synthesis, versus the time on stream (TOS) are given in Figure 6. TOS averaged FT-olefin performance parameters related to each catalyst are summarized Table 5.

Conversion profiles given in Figure 6 show that the initial conversion measured on each catalyst was in the 85%–95% range. Nevertheless, as the time on stream increased, loss of activity, although at different levels, was observed on all catalysts. The decrease in activity by time was the lowest on 15Fe0.3Mn0.5Cu catalysts. It was observed that the use of pentane as impregnation agent was effective in preventing activity loss in Cu promoted catalysts. Similar effect of pentane was also observed on 15Fe0.3Mn0.5K catalyst. The decrease in CO conversion occurred both faster and at a higher degree on 15Fe0.3Mn0.5K-w relative to that observed on other catalysts. However, pentane affected the structure of 15Fe0.3Mn0.5K catalyst such that the rate of deactivation was slower and the final conversion was higher compared to its water based counterpart. The effect of pentane on catalysts achieving higher CO conversions is due to its nonpolar nature enabling better metal-support interaction, as described earlier. Combining Cu and K as promoters on 15Fe0.3Mn catalysts led to a CO conversion profile vs. TOS that fell right in the middle of Cu promoted and K promoted catalysts.

The deactivation observed on catalysts could be due to the loss of active sites as a result of coke deposition, agglomeration or reoxidation of active iron carbide phases during the course of the reaction [42,43]. Iron carbides act as active phase in FT synthesis [51, 53, 54]. Cu as a promoter is reported to enhance reducibility and facilitate carburization, hence improve FT synthesis activity [34,37]. The distinct features of both iron carbide phases can be clearly observed in XRD profiles of the Cu promoted catalysts as given in Figure 4. While the presence of iron carbide phases explains the high activity of these catalysts, the slight decrease in CO conversion can be attributed to reoxidation of iron carbides to Fe_3_O_4_. The reoxidation to metal oxides was highly present on K promoted catalysts. K promoter facilitates the dissociation of CO on catalyst surface as significantly as it hinders the adsorption of H_2_ [37,55]. This impedes effective reduction of surface iron species [48]. Thus, the presence of oxide phases in the XRD profiles of K promoted catalysts in Figure 4 align with the literature predicted behavior of K, and explain the low activity of the catalysts. When Cu and K act as double promoter, the combined effects of both metals result in a mid-level CO conversion. For Cu or K promoted catalysts, the effect of pentane agent in leading to higher conversions can be explained by the presence of more emphasized carbide phases in pentane-used-catalysts compared to their water-used counterparts.

The LOs selectivity of the catalysts, represented as C_2_^=^-C_4_^=^ in Table 5, also varied based on promoter and impregnation agent types. The highest LOs selectivity was observed on K promoted catalysts. K enhances olefin selectivity and suppresses paraffins production [37,56]. Therefore, the paraffin selectivity was also the lowest on K promoted catalysts, leading to high olefin-to-paraffin (O/P) ratios. However, K is also reported to increase the production of heavy hydrocarbons [37].

On Cu promoted catalysts, almost similar selectivities were observed for LOs and paraffins. This can be attributed to higher rates of secondary hydrogenation reactions on these catalysts facilitated by Cu promotion [37]. Yet, Cu was effective in shifting product distribution towards gas hydrocarbons.

Cu-K double promotion provided both the high activity of Cu promotion and the high LOs-low paraffins selectivity of K promotion. On the other hand, in Cu-Ni double promoted catalyst, Ni, as expected [20, 21], shifted HC distribution towards methane and enhanced Cu promotion on secondary hydrogenation reactions leading to low LOs selectivity.

In FT synthesis, a high correlation between pore geometry and C_5+_ production exists [57, 58]. It is apparent that this correlation is valid for catalysts in this study comparing the pore size values obtained by N_2_ adsorption listed in Table 3 with C_5+_ formation of related catalysts; as the pore size got larger, C_5+_ production of catalysts increased.

Considering CO_2_ selectivity in Table 5 with the H_2_/CO usage ratio in Figure 7, the extent of water gas shift (WGS) on each catalyst can be identified [53, 59]. Although the CO_2_ selectivity values do not vary significantly based on promoter or impregnation agent type, reported values in Figure 7 state that 15Fe0.3Mn0.5Cu-p and 15Fe0.3Mn0.5Cu0.5Ni-w catalysts were slightly more effective in suppressing WGS activity. This is an expected result as K promoter is known to increase WGS activity of the catalysts [11,52]. In addition to WGS activity, H_2_/CO usage ratio also aligns well with LOs selectivity and O/P ratio. Low values indicate more double bond formation which align with high LOs selectivities and O/P ratios observed on 15Fe0.3Mn0.5K-w, 15Fe0.3Mn0.5K-p and 15Fe0.3Mn0.5Cu0.5K-w catalysts.

For benchmarking purposes, FT-olefin performances of plain 15Fe-w, and Mn promoted 15Fe0.3Mn-w and 15Fe0.3Mn-p catalysts were also tested and summarized in Table 5. These results reveal that Cu and/or K addition and use of pentane enhances catalyst performance. Cu is significant in increasing catalyst activity, whereas K is significant in enhancing LOs selectivity and O/P. The results of Cu promoted and K promoted catalysts in this study were also compared with their counterparts in the study by Mangaloglu et al. [60]. In that study, ZSM-5 supported 9Fe0.45Mn0.45Cu, 9Fe0.45Mn0.45K and 9Fe0.45Mn0.45Cu0.45K catalysts were subjected to FT synthesis at 280 °C and 19bar. Although the temperature was slightly lower than the one used in this study, the pressure was almost doubled; and there is a slight difference in active metal/promoter compositions of both studies. While the CO conversion of the catalysts analyzed by Mangaloglu et al. did not exceed 40%, LOs selectivities were well below the values reported in this study. Therefore, this comparison is critical in pointing out the importance of metal concentrations and support selection to control product distribution in FTS, and the effectiveness of HT-CPA screening in catalyst discovery.

### 3.7. HT-CPA vs P-CPA results

As described earlier, HT-CPA allows for fast catalytic performance screening of large numbers of different catalysts. The fact that HT-CPA system operates at only atmospheric pressure limits its capability in catalyst development to only prediction of candidate catalysts for reactions such as FT that requires high pressures. Nevertheless, this does not devaluate the quality or the quantity of data obtained in HT-CPA.

In this study, HT-CPA tests were performed on 40 different catalysts to determine the candidates that could show relatively higher performance. Based on the performance criteria defined in this study for reaction at atmospheric pressure, 15Fe0.3MnCu-w, 15Fe0.3Mn0.5Cu-p and 15Fe0.3Mn0.5K-p were regarded as the top performing catalysts followed by 15Fe0.3Mn0.5Cu0.5K-w. On the other hand, 15Fe0.3Mn0.5Cu0.5Ni-w catalyst was identified as the worst performing catalyst for FT-olefin synthesis.

As summarized in Table 6, P-CPA results (Table 5) for FT-olefin synthesis were aligned with HT-CPA predictions (Table 1). 15Fe0.3Mn0.5Cu0.5Ni-w catalyst displayed the poorest FT-olefin performance under high pressure and high temperature operation. Highest CO conversions and olefin yields were observed on 15Fe0.3Mn0.5Cu-p (93.4%) and –w (88.4%) catalysts in P-CPA. These catalysts were also the most stable catalysts. K comprising catalysts, especially 15Fe0.3Mn0.5K-p, exhibited the high O/P ratios. These results prove that coupling HT-CPA analysis (at atmospheric pressure) with P-CPA tests can be utilized effectively in FT-olefin catalysts discovery.

## 4. Conclusion

In this study, the effect of promoter and impregnation agent on FT-olefin performances of 40 different α-Al_2_O_3_ supported Fe (15 wt.%)-Mn (0.3 wt.%) based catalysts were investigated. Cu, Ni and K were selected to investigate the effect of promoter; deionized water and n-pentane were selected to investigate the effect of impregnation agent. Six catalysts were chosen regarding their FT-olefin performances in HT-CPA, ensuring the sampling from high and low performance catalysts. These six catalysts were subjected to high pressure reaction, to observe their performance at realistic FTS conditions. Out of these 6 catalysts, the higher performing 4 catalysts were also subjected to the characterization analysis to elucidate activity-structure relation. Conclusions regarding these analyses can be summarized as follows:

- Cu promotion led to high and stable FTS activity (88% and 93% CO conversion for water and pentane, respectively).
- Although K promoter led to a decrease in activity, highest light olefins selectivity and olefin-to-paraffin ratios (6.71 and 7.03 for water and pentane, respectively) were obtained on 15Fe0.3Mn0.5K catalysts. To the best of our knowledge, there is yet no reported supported Fe-Mn-K catalysts with a better cumulative (activity, selectivity, O/P) FT-olefin performance than that of the Fe-Mn-K catalyst analyzed in this study.
- A synergistic interaction between Cu and K was observed on 15Fe0.3Mn0.5Cu0.5K catalyst. The stability and activity loss due to K promotion was balanced by Cu, keeping CO conversion at ca. 82%. On the other hand, K suppressed secondary hydrogenation reactions, leading to high light olefins selectivity (approximately 42%) and olefin-to-paraffin ratio (4.46).
- Agent selection for impregnation slightly altered the catalyst performances. The nonpolar nature of n-pentane, compared to the highly polar water, enhanced metal-support interaction during catalyst synthesis, leading to higher CO conversions. These results are important in revealing the enhancing effect of n-pentane aided impregnation for the first time in FT-olefin catalyst literature.
- To the best of our knowledge, this study presents the successful coupling of high-throughput-conventional catalyst screening methods for the fırst time in literature on FT-olefin catalysts. This not only decreases the time and resources spent on catalyst discovery, but also enables for screening a wide variety of options highly increasing the probability of discovering the optimum catalyst formulation.

## Figures and Tables

**Table 1:** Prepared catalysts and the comparative HT-CPA analysis results. Reduction conditions: T = 350 °C, P = 1 bar, H_2_:N_2_= 1:1. Reaction conditions: T = 310°C, P= 1 bar, H_2_:CO = 2:1. Color-coding represents the change from desired to undesired results with the change from green to red color.

	Method	Water				n-pentane				Water				n-pentane				
Performance indicator	wt.%	15Fe0.3MnxCu	15Fe0.3Mn0.5CuxNi	15Fe0.3Mn0.5CuxK	15Fe0.3MnxK	15Fe0.3MnxCu	15Fe0.3Mn0.5CuxNi	15Fe0.3Mn0.5CuxK	15Fe0.3MnxK	15Fe0.3MnxCu	15Fe0.3Mn0.5CuxNi	15Fe0.3Mn0.5CuxK	15Fe0.3MnxK	15Fe0.3Mn	15Fe0.3Mn0.5CuxNi	15Fe0.3Mn0.5CuxK	15Fe0.3MnxK	Performance indicator
CO conversion	0.5																	CO_2_ production
	1																	
	1.5																	
	2																	
	2.5																	
Total C_2_^=^-C_4_^=^	0.5																	CH_4_ production
	1																	
	1.5																	
	2																	
	2.5																	
C_2_^=^-C_4_^=^/CH_4_ free HC	0.5																	CH_4_/CH_4_ free HC
	1																	
	1.5																	
	2																	
	2.5																	

**Table 2:** Final grades comparing FT-olefin performance of the catalysts. Reduction conditions: T= 350 °C, P = 1 bar, H_2_:N_2_ = 1:1. Reaction conditions: T = 310 °C, P = 1 bar, H_2_:CO = 2:1. Color-coding represents the change from desired to undesired results with the change from green (100 points) to red (0 points) color.

Method	Water				n-pentane			
wt.%	15Fe0.3MnxCu	15Fe0.3Mn0.5CuxNi	15Fe0.3Mn0.5CuxK	15Fe0.3MnxK	15Fe0.3MnxCu	15Fe0.3Mn0.5CuxNi	15Fe0.3Mn0.5CuxK	15Fe0.3MnxK
0.5	96	30	90	79	100	28	82	94
1	88	25	83	76	92	36	70	86
1.5	91	27	74	79	84	10	73	87
2	72	15	80	84	78	11	85	89
2.5	73	22	74	81	77	0	85	89

**Table 3:** Textural properties of fresh (calcined) catalysts.

Catalyst	BET surface area (m^2^/g)	Pore volume (10^−3^cm^3^/g)	Average pore diameter (nm)	Micropore volume (10^−3^cm^3^/g)	External surface area (m^2^/g)
15Fe0.3Mn–w	19.10	4.03	8.43	0.02	18.74
15Fe0.3Mn0.5Cu–w	19.56	3.34	6.83	0.23	18.84
15Fe0.3Mn0.5K–w	8.98	3.43	15.28	1.68	5.68
15Fe0.3Mn–p	13.30	3.82	11.50	0.81	11.61
15Fe0.3Mn0.5Cu–p	9.95	2.66	10.70	1.58	6.91
15Fe0.3Mn0.5K–p	8.92	3.72	16.69	1.69	5.60

**Table 4:** Crystallite size of Fe_2_O_3_ phase on fresh (calcined) catalysts.

Catalyst	Fe_2_O_3_ crystallite size (nm)
15Fe0.3Mn0.5Cu-w	10.47
15Fe0.3Mn0.5Cu-p	14.38
15Fe0.3Mn0.5K-w	17.13
15Fe0.3Mn0.5K-p	20.58

**Table 5:** TOS averaged FT-olefin performance of catalysts (Reduction conditions: T = 350 °C, P = 1 bar, H_2_:N_2_= 1:1, Reaction conditions: T = 310 °C, P = 10 bar, H_2_:CO = 2:1).

Catalyst	X_CO_ %	S_CO2_ (% C)	S (% C) Total HC	S (% C) coke/heavier HC	Hydrocarbon distribution (C %)					C_2_^=^-C_4_^=^ Yield (gC/gFe.s) (x10^−4^)	FT yield (mol CO converted/gFe.s) (x10^−5^)
					CH_4_	${\text{C}}_{2}^{=}-{\text{C}}_{4}^{=}$	${\text{C}}_{2}^{\text{o}}-{\text{C}}_{4}^{\text{o}}$	C_5+_	O/P		
15Fe-w	39.7	18.5	81.5	31.1	38.0	26.3	22.9	11.6	1.26	0.40	1.64
15Fe0.3Mn-w	51.8	23.0	77.0	39.7	40.1	32.3	19.4	7.9	1.66	0.60	1.80
15Fe0.3Mn-p	72.9	25.6	74.4	42.5	34.7	29.1	28.2	7.9	1.03	0.73	2.37
15Fe0.3Mn0.5Cu-w	88.4	39.2	60.8	23.6	33.0	33.2	28.7	5.1	1.16	1.13	2.81
15Fe0.3Mn0.5Cu-p	93.4	37.0	63.0	30.1	30.4	28.2	31.3	9.6	0.90	0.97	2.87
15Fe0.3Mn0.5K-w	49.6	43.7	56.3	66.9	22.8	49.8	7.4	18.9	6.71	0.37	0.64
15Fe0.3Mn0.5K-p	55.8	43.5	56.5	67.6	24.8	46.6	6.6	20.2	7.03	0.39	0.71
15Fe0.3Mn0.5Cu0.5Ni-w	71.7	36.8	63.2	23.1	54.0	11.5	30.4	4.1	0.38	0.29	2.42
15Fe0.3Mn0.5Cu0.5K-w	81.8	40.8	59.2	70.0	29.2	42.2	9.4	18.7	4.46	0.52	1.02

**Table 6:** Summary of HT-CPA evaluation and P-CPA outcomes.

Method	Water				n-pentane	
Catalyst	15Fe0.3Mn0.5Cu	15Fe0.3Mn0.5Cu0.5Ni	15Fe0.3Mn0.5Cu0.5K	15Fe0.3Mn0.5K	15Fe0.3Mn0.5Cu	15Fe0.3Mn0.5K
HT-CPA evaluation	96	30	90	79	100	94
P-CPA outcome	Highest C_2_^=^-C_4_^=^ yield	Low C_2_^=^-C_4_^=^ selectivity and O/P, high CH_4_ selectivity	High CO conversion, C_2_^=^-C_4_^=^ selectivity and O/P	Highest C_2_^=^-C_4_^=^ selectivity	Highest CO Conversion, high C_2_^=^-C_4_^=^ yield	High C_2_^=^-C_4_^=^ selectivity, highest O/P
